# Supplementary material for: Conditional chemoconnectomics (cCCTomics) as a strategy for efficient and conditional targeting of chemical transmission
Source: eLife. 2024 Apr 30;12:RP91927. doi: 10.7554/eLife.91927 (PMC11060718; doi:10.7554/eLife.91927)
Supplement: Figure 3—source data 3. [file elife-91927-fig3-data3.zip › Figure 3 source data3 part 2.zip!/Figure 3-Source data 3-part2/1025-13-CRISPResso_Report_ack040l3.zip!/CRISPResso_on_1025_15/1c.Alignment_barplot.pdf]

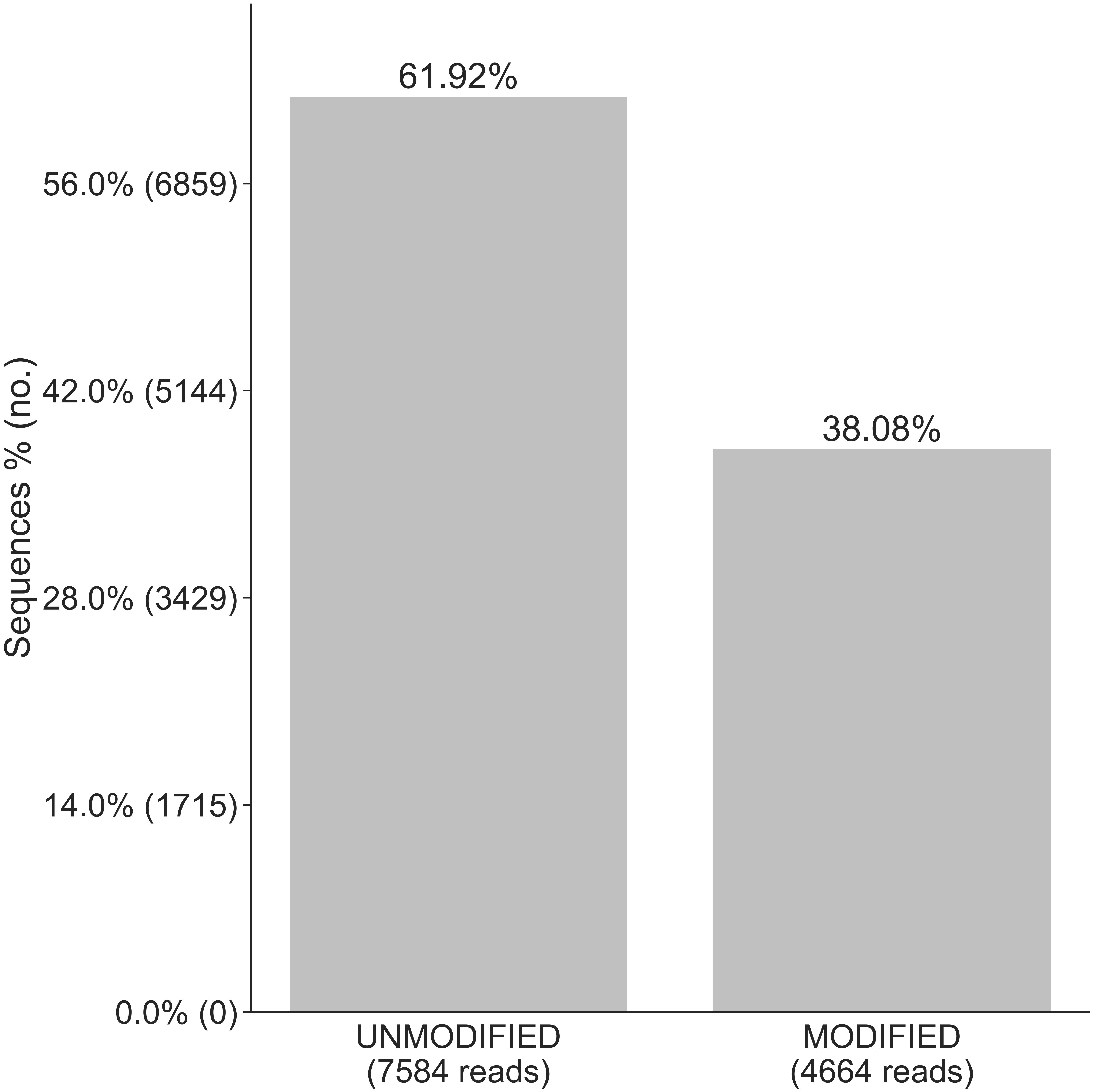
Sequences % (no.)
61.92%
38.08%
56.0% (6859)
42.0% (5144)
28.0% (3429)
14.0% (1715)
0.0% (0)
UNMODIFIED
(7584 reads)
MODIFIED
(4664 reads)
